# The influence of human settlement on the distribution and diversity of iron-oxidizing bacteria belonging to the Gallionellaceae in tropical streams

**DOI:** 10.3389/fmicb.2014.00630

**Published:** 2014-11-24

**Authors:** Mariana P. Reis, Marcelo P. Ávila, Patrícia S. Costa, Francisco A. R. Barbosa, Hendrikus J. Laanbroek, Edmar Chartone-Souza, Andréa M. A. Nascimento

**Affiliations:** ^1^Departamento de Biologia Geral, Instituto de Ciências Biológicas, Universidade Federal de Minas GeraisBelo Horizonte, Brazil; ^2^Department of Microbial Ecology, Netherlands Institute of Ecology (NIOO – KNAW)Wageningen, Netherlands; ^3^Institute of Environmental Biology, Utrecht UniversityUtrecht, Netherlands

**Keywords:** iron-oxidizing bacteria, 16S rRNA, *Gallionella*, *Sideroxydans*, freshwater sediment, mining, metals

## Abstract

Among the neutrophilic iron-oxidizing bacteria (FeOB), *Gallionella* is one of the most abundant genera in freshwater environments. By applying qPCR and DGGE based on 16S rRNA gene-directed primers targeting Gallionellaceae, we delineated the composition and abundance of the Gallionellaceae-related FeOB community in streams differentially affected by metal mining, and explored the relationships between these community characteristics and environmental variables. The sampling design included streams historically impacted by mining activity and a non-impacted stream. The sediment and water samples harbored a distinct community represented by *Gallionella, Sideroxydans*, and *Thiobacillus* species. Sequences affiliated with *Gallionella* were exclusively observed in sediments impacted by mining activities, suggesting an adaptation of this genus to these environments. In contrast, *Sideroxydans*-related sequences were found in all sediments including the mining impacted locations. The highest and lowest relative frequencies of Gallionellaceae-related FeOB were associated with the lowest and highest concentrations of Fe, respectively. The data enclosed here clearly show distinct species-specific ecological niches, with *Gallionella* species dominating in sediments impacted by anthropogenic activities over *Sideroxydans* species.

## Introduction

Iron (Fe), the second most abundant metal in the lithosphere (Lutgens and Tarbuck, 2000), plays an essential role in biological processes such as photosynthesis, N_2_ fixation, and respiration (Andrews et al., 2003). Due to the importance of Fe in biogeochemical redox cycles and its role as a potential microbial energy source, considerable research efforts have been invested in aiming to reveal the nature and numbers of ferrous iron Fe(II)-oxidizing bacteria (FeOB) in the environment (for example Ghiorse, 1984; Emerson and Weiss, 2004; Wang et al., 2009, 2011).

Lithotrophic FeOB are able to compete with abiotic Fe(II) oxidation in the presence of O_2_, thereby increasing the rate of Fe oxidation when compared with the strictly abiotic oxidation (Hallbeck et al., 1993; Hallbeck and Pedersen, 1995; Sogaard et al., 2001; Druschel et al., 2008). Moreover, these bacteria have been shown to play a role in the cycling of carbon, nutrients, and other metals (Lovley, 2000). However, for a long time the knowledge about lithotrophic FeOB was limited to acidophilic bacteria (Harrison, 1984; Pronk and Johnson, 2002; Auernik et al., 2008). It is only over the past 20 years that microaerophilic, neutrophilic FeOB have been shown to oxidize Fe at oxic-to-anoxic boundaries under circumneutral pH conditions (Emerson and Floyd, 2005; Emerson et al., 2010; Hedrich et al., 2011), reaching up to 60% of the total Fe oxidation (Emerson and Moyer, 2002).

Whereas data on the phylogenetic diversity and eco-physiological properties of acidophilic FeOB are widely available (Leduc and Ferroni, 1994; Pronk and Johnson, 2002), particularly from mining-impacted environments (Johnson and Hallberg, 2003), a poorer knowledge scenario applies for neutrophilic FeOB. With respect to phylogenetic diversity, only a few species of neutrophilic FeOB have been retrieved from circumneutral pH environments to date; for example, *Gallionella ferruginea, Sideroxydans lithotrophicus*, and *Mariprofundus ferrooxydans*, the first two belonging to the Betaproteobacteria and the last one to the Zetaproteobacteria (Emerson et al., 2010; Hedrich et al., 2011; McBeth et al., 2011). With respect to eco-physiological properties, the studies published so far have focused on wetland soils and sediments (Wang et al., 2009, 2011), freshwater environments (Lin et al., 2012), marine sediments (McAllister and Milioli, 2000), and small streams (Fleming et al., 2014). However, knowledge about the effect of mining activity on the phylogenetic diversity and ecological properties of neutrophilic FeOB is lacking.

To gain insight into this knowledge gap we investigated the phylogenetic diversity and ecological properties of Gallionellaceae-related FeOB present in streams historically impacted by mining activity, and in a stream not impacted as reference. Gallionellaceae-related FeOB are known to be abundant in freshwater environments (Hedrich et al., 2011). Moreover, because the streams studied exhibited a wide variation in the concentrations of metals and inorganic nutrients, we also studied the relationship of these neutrophilic bacteria with environmental physicochemical variables.

The data presented provide insights into the ecology and phylogeny of Gallionellaceae-related FeOB in metal-rich environments. The streams analyzed may also be interesting sources of neutrophilic FeOB enabling extending the current knowledge about their phylogenetic diversity and ecology.

## Materials and methods

### Study area

Five sampling sites had been chosen in the Iron Quadrangle region (Minas Gerais state, Brazil), which is extremely rich in ores and has been the scene of gold extraction since colonial times, and of mining for iron and manganese since the nineteenth century. Three sampling sites were chosen in mining-impacted streams, i.e., in the Mina stream (MS; 19°58′46.80″S and 43°49′17.07″W), in the Tulipa stream (TS; 19°59′08.1″S and 43°28′15.2″W), and in the Carrapatos stream (CS; 19°58′15.4″S and 43°27′50.7″W), and two sites in a non-impacted stream (19°59′12.1″S and 43°29′27.5″W) which did not experience mining activities. The sampled sites in the non-mining-impacted stream were designed S1 and S2. With the exception of the Mina stream, the streams do not have formal names. Two of them are popularly known as Tulipa and Carrapatos (Figure S1). An interesting characteristic of these streams is their circumneutral pH. Although the dissolution of minerals can cause the production of acidic wastes and lead to lower pH values (Smedley and Kinniburgh, 2002), the abundance of dolomite in the ores from the Iron Quadrangle region neutralizes the acids with consequent buffering of the pH (Thomas et al., 2000; Cidu et al., 2009; Varejão et al., 2011; Reis et al., 2013).

Samples were aseptically collected in August 2012 (dry season), and taken from bulk water (5L) and billowy sediment at a depth of 15 and 5 cm below the respective surfaces, respectively. Sediment was sampled at five random spots located in line, 1 m from each other. The sediment samples were then pooled into a single sample corresponding to the study site, and transported to the laboratory within 4 h on ice and stored at −20°C until further processing. The samples were labeled by the name of the stream followed by W (water) or S (sediment), e.g., CW (Carrapatos stream water) and CS (Carrapatos stream sediment).

To assess the bulk water conditions of the streams, physicochemical variables were measured. Temperature, pH, and dissolved oxygen (DO) were measured *in situ* with a Hydrolab model DS5X multi-parameter probe. Total nitrogen (TN), total phosphorus (TP), nitrite (NO^−^_2_-N), nitrate (NO^−^_3_-N), and soluble reactive phosphorus (PO^3−^_4_-P) were measured as previously described by Mackereth et al. (1978). The concentration of ammonium (NH^+^_4_-N) was measured according to Koroleff (1976). The readings were performed with a Shimadzu model UV 1700 spectrophotometer. Metal and metalloid concentrations of bulk water and sediment of all the samples were determined by using inductively coupled plasma-optical emission spectrometry (ICP-OES, Optima 7300 DV, PerkinElmer).

### DNA extraction and PCR-denaturing gradient gel electrophoresis (DGGE)

Total DNA was extracted from all the sediment samples (10 g wet weight) and from the Carrapatos stream bulk water sample (5L) using the UltraClean Mega Prep soil DNA kit and UltraClean Water DNA kit (MoBio Laboratories), respectively, according to the manufacturer's instructions. Quantification and quality of total DNA were determined using the equipment Agilent 2100 Bioanalyzer, again according to the manufacturer's instructions.

Wang et al. (2009) successfully applied 16S rRNA gene-based approaches using Gallionellaceae-related primers, to describe the FeOB community structure. PCR-DGGE of 16S rRNA gene was performed by a nested PCR using first the Gallionellaceae-related primers set 122F (5′-ATATCGGAACATGTCCGG-3′) and 998R (5′-CTCTGGAAACTTCCTGAC-3′) (Wang et al., 2009), followed by nested PCR using the primers set 341F/GC (5′-CCTACGGGAGGCAGCAG-3′) and 907R (5′-CCGTCAATTCMTTTGAGTTT-3′), specific for the Bacteria domain (Muyzer et al., 1993), according to Wang et al. (2011). The final PCR products were separated by DGGE in a 6% polyacrylamide gel with a vertical gradient of 30–60% of formamide and urea denaturants. The running conditions were 80 V at a constant temperature of 60°C for 18 h.

### DGGE-band sequencing and phylogenetic analysis

The phylogenetic assignment of Gallionellaceae-related FeOB from samples was determined by excising DGGE bands, which were eluted in 20 μl of sterile MilliQ water overnight at 6°C, and sequencing, using the same primers set 341F and 907R, in the ABI Prism 3130 DNA sequencer (Applied Biosystems, Foster City, Calif.). Partial sequences were checked for quality, aligned, and edited to produce a consensus using the Linux programs Phred/Phrap/Consed (http://www.phrap.org/phredphrapconsed.html). The Bellerophon program (Huber et al., 2004) was used to detect and omit chimeric DNAs. Sequences were compared with available databases using the BLASTn search tool from GenBank (http://www.ncbi.nlm.nih.gov/) and the Greengenes Project (http://greengenes.lbl.gov/) to identify the closest relatives of the sequences.

Sequence alignment and phylogenetic relationships were inferred with ARB (Ludwig et al., 2004; Pruesse et al., 2007) using the neighbor-joining algorithm (http://www.arb-home.de). The bootstrap consensus tree inferred from 500 replicates (Felsenstein, 1985) was taken to represent the evolutionary history of the taxa analyzed.

Bands at identical positions in the DGGE gel are not necessarily derived from the same species nor do distinct bands indicate the same bacterium (Emerson and Moyer, 1997). Thus, whenever possible, we sequenced at least two bands at similar positions on the DGGE gel. The nucleotide sequences (288 bp) generated were deposited in the GenBank database with accession numbers KJ363887 to KJ363908.

### Quantitative PCR (qPCR)

For each sediment and Carrapatos bulk water sample we estimated the abundance of the 16S rRNA gene associated with Gallionellaceae-related FeOB and with the Bacteria domain using an ABI PRISM 7900HT sequence detection system (Applied Biosystems, Foster City, CA). The primer sets 628F (5′-GBMAGGCTAGAGTGTAGC-3′) and 998R (Wang et al., 2009), and 338F (5′-TACGGGAGGCAGCAG-3′) (Raskin et al., 1994) and 518R (5′-ATTACCGCGGCTGCTGG-3′) (Muyzer et al., 1993) were used for the amplification of 16S rRNA genes from the Gallionellaceae-related FeOB and the Bacteria domain, respectively. Reaction conditions for Gallionellaceae-related FeOB and the Bacteria domain were as described by Wang et al. (2011) and Reis et al. (2013), respectively. Each sample was run in triplicate and a negative control was included for each analysis. Relative frequency values of Gallionellaceae-related 16S rRNA genes were calculated for each sample (McCune and Grace, 2002). Positive controls consisted of a mix of all DNA from samples, which were amplified using the primer sets specific for Gallionellaceae-related FeOB and the Bacteria domain, respectively. The standard curves for the primer sets generated slopes of −3.39 and −3.22, respectively, and the *R*^2^ values were greater than 0.99 for both curves (Figure S2). Bacteria domain qPCR exhibited a wide range of *Ct-values* ranging from 13 to 25. On the other hand, lower variation of *Ct*-values was obtained for Gallionellaceae-related FeOB (from 24 to 27) (Figure S3).

### Data analysis

For DGGE data, the presence or absence of co-migrating bands was converted to a binary matrix (0/1). A dendrogram was calculated using Jaccard coefficient of similarity and the un-weighted pair-group method with arithmetic averages (UPGMA). Analysis of data was performed using the software PAST (Paleontological Statistics Software Package) (Hammer et al., 2001).

To correlate the distribution of samples according to environmental parameters, we performed a Principal Component Analysis (PCA). After standardization of environmental data (by subtracting the mean from each observation and dividing by the corresponding standard deviation), PCA was obtained using the *rda* function in the Vegan library program implemented in R (<http://www.r-project.org/>). This statistical technique is frequently used to analyze a data table representing the environmental variables of each sample. Thus, the important information is extracted from the data table and is expressed as a set of new orthogonal variables called principal components. PCA also demonstrates the pattern of similarity of the samples and the variables by plotting them as points in maps (Jolliffe, 2002).

## Results

### Environmental characterization

In an attempt to characterize the environmental conditions and correlate them with the dynamics of the neutrophilic FeOB community, we analyzed the physicochemical characteristics of the bulk stream water, as well as the metal and metalloid concentrations in bulk water and in sediment samples from the five stream locations.

Although the pH values of the bulk water samples varied largely, all were less than 1 unit different from neutral (Table 1). Values of conductivity showed also considerable variation with the highest value in the MW sample, and the lowest in the SW1 and SW2 samples. Dissolved oxygen (DO) concentrations ranged from 3.62 (TW) to 12.52 (CW) mg O_2_ l^−1^. The water samples varied widely with regard to nitrogen and phosphorus concentrations. Mining-impacted streams showed much higher NH^+^_4_-N concentrations than the non-impacted stream (SW1 and SW2). Moreover, NO^−^_3_-N values displayed by the Mina stream were remarkably higher than the values shown by all the other streams. Mina stream showed also higher values of NO^−^_2_-N. Moreover, Tulipa stream exhibited the same concentrations of NO^−^_2_-N and NO^−^_3_-N. Total phosphorus concentrations allowed categorization of the streams as eutrophic (Mina stream), mesotrophic (Carrapatos stream), oligotrophic (non-mining-impacted stream), and ultraoligotrophic (Tulipa stream) (Table 1).

**Table 1:** **Physicochemical characteristics of the bulk water samples**.

	**CW**	**TW**	**MW**	**SW1**	**SW2**
**GENERAL CHARACTERISTICS**					
pH	7.91	6.53	6.2	6.66	6.5
Conductivity (μS cm^−1^)	227	1500	2151	13	13
Temperature (°C)	17.30	22.98	18	18.49	20
DO (mg O_2_ l^−1^)	12.52	3.62	9.1	12.11	12.11
**CHEMICAL CHARACTERISTICS**					
Total P (μg l^−1^)	63.72	1.48	77.6	10.64	10.64
PO_4_-P (μg P l^−1^)	2.28	0.19	2.3	2.31	2.31
NH^+^_4_-N(μg N l^−1^)	122.65	1600	829.5	25.1	25.1
NO_2_-N (μg N l^−1^)	0.51	12.53	161.3	0.2	0.2
NO_3_-N (μg N l^−1^)	6.0	13.33	3103.8	4.57	4.57

*CW, water from Carrapatos stream; TW, water from Tulipa stream; MW, water from Mina stream; SW1 and SW2, water from sites 1 and 2 of the non-impacted mining stream*.

Metal and metalloid concentrations of water and sediment samples varied widely (Table 2). The CS sample showed strikingly high concentrations of Fe, but also considerable amounts of Mn and Mg. Interestingly, Fe concentration found in the sediments from the other mining-impacted streams (TS and MS) was lower than the sediments obtained from non-impacted stream (S1 and S2). The MS was rich in As, Cu, Zn, and Mn (Table 2).

**Table 2:** **Metal and metalloids concentrations of water and sediment samples (mg l^−1^ in water, mg kg^−1^ in sediments)**.

**Metal and metalloids**	**CONAMA**		**CW**	**CS**	**TW**	**TS**	**SW1**	**SS1**	**SW2**	**SS2**	**MW**	**MS**
	**water^a^**	**sediment^b^**										
As	0.5	5.9	0.14	<1.25	<0.05	<1.25	<0.05	<1.25	<0.05	<1.25	<0.1	297.1
Cr	1	37.3	<0.1	12.75	<0.1	<2.5	<0.1	<2.5	<0.1	6.25	<0.1	17.3
Cu	1	35.7	<0.05	5.75	<0.05	8.75	<0.05	9.5	<0.05	18.75	0.19	387.7
Fe	15	*NE*	2.13	21,850	<0.05	437.5	0.11	2136	0.11	5238	0.52	492.8
Mg	*NE*	*NE*	14.84	1416	285.9	462.5	0.8	378	0.8	314	*NT*	*NT*
Mn	1	*NE*	1.04	2319	0.59	197.75	<0.05	1405	<0.05	392.5	1.45	1285
Ni	2	18	<0.05	13.25	<0.05	2.5	<0.05	7.5	<0.05	6.25	<0.1	9
Pb	0.5	35	<0.1	3.5	<0.1	9.5	<0.1	2.75	<0.1	9	*NT*	8.7
Zn	5	123	<0.05	24.5	<0.05	2.25	<0.05	5.75	<0.05	14.75	0.2	180.9

*NT, not tested*.

*NE, not established*.

a *Conselho Nacional do Meio Ambiente—Brazil (CONAMA) resolution 430/11*.

b *Conselho Nacional do Meio Ambiente—Brazil (CONAMA) resolution 344/04*.

*CW and CS, water and sediment from Carrapatos stream; TW and TS, water and sediment from Tulipa stream; MW and MS, water and sediment from Mina stream; SW1, SW2, SS1, and SS2 water and sediment from sites 1 and 2 of the non-impacted mining stream, respectively*.

**Figure 1:**
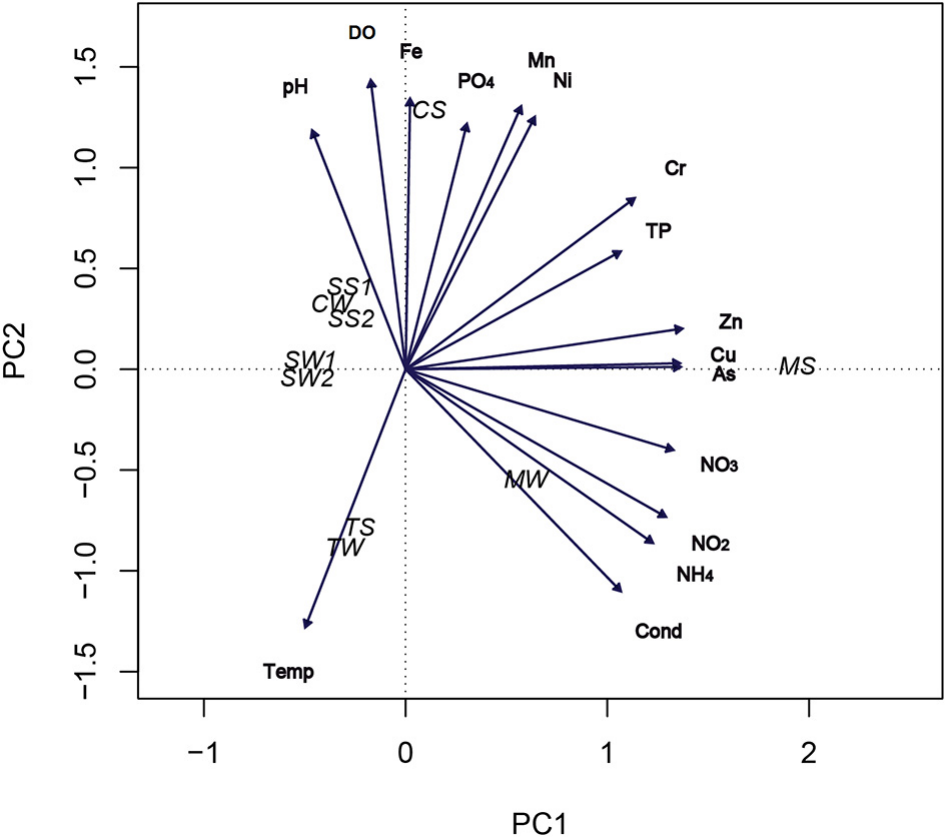
**PCA ordination biplot of sample locations according to environmental parameters**. CW, Carrapatos water; TW, Tulipa water; MW, Mina water; SW1, Site 1 water; and SW2, Site 2 water; CS, Carrapatos sediment; MS, Mina sediment; SS1, Site 1 sediment; SS2, Site 2 sediment; TS, Tulipa sediment.

PCA analysis revealed that the mining-impacted MS, CS, and TS sediment samples were clearly separated from each other, whereas the SS1, SW1, SS2, SW2, and CW samples were closer to each other (Figure 1). The MS sample was largely determined by the first axis (PC1) of the PCA biplot with positive values for inorganic nitrogen, Zn, Cu, and As. The MW sample was also determined by PC1 axis, with positive values for conductivity, NH^+^_4_-N and NO^−^_2_-N. The CS, TS, and TW samples were mainly determined by the second axis (PC2) with the variables PO_4_-P, Mn, Fe, DO, and temperature. The first four variables of this axis were positively correlated to the CS sample, and negatively correlated to the TS and TW samples. The opposite was observed for the TS and TW samples. The data show a clear separation between the mining-impacted and non-impacted sediment samples, with the bulk water sample CW more closely resembling the non-impacted sediments, whereas the MW and TW bulk water samples were closer to their respective sediment samples MS and TS. The first two axes of the PCA accounted for 72% of the total variance of sample distribution (Figure 1).

### Analysis of DGGE banding patterns

**Figure 2:**
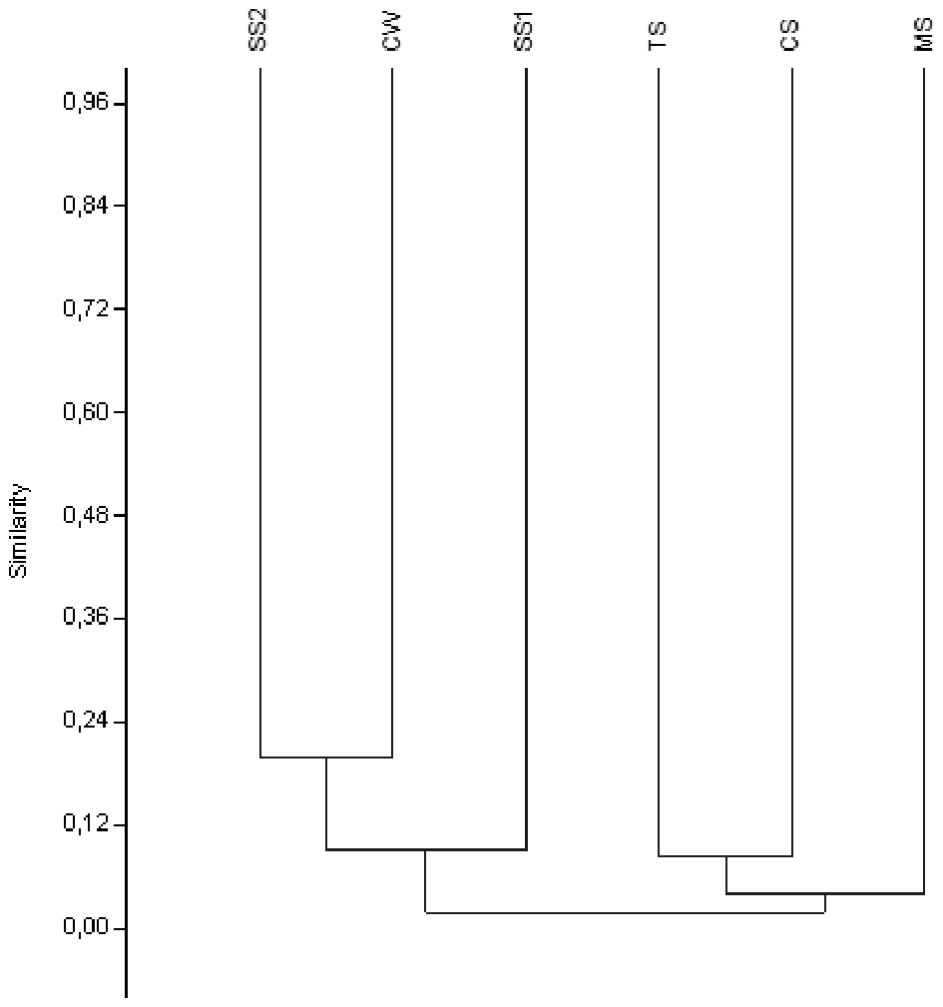
**UPGMA cluster analysis of the Gallionellaceae-related FeOB band sequences from the samples**. CW, Carrapatos water; CS, Carrapatos sediment; MS, Mina sediment; SS1, Site 1 sediment; SS2, Site 2 sediment; TS, Tulipa sediment.

Because the highest concentration of Fe among the bulk water samples was derived from the Carrapatos stream, we decided to focus the molecular analyses of water samples to water sample of this stream. Most DGGE bands representing Gallionellaceae-related FeOB were unique for a given sample, only a few were shared between two samples, and none was common to all samples (Figure S4). The CS sample produced the largest number of bands, whereas the other samples exhibited the same number of bands (Figure S4). The UPGMA cluster analysis revealed a distinct bacterial community composition of the samples analyzed (Figure 2). The Gallionellaceae-related FeOB community from the MS sample was most dissimilar, whereas the communities retrieved from SS1, CW, and SS2 samples were the least dissimilar (Figure 2).

### Phylogenetic assignment of gallionellaceae-related FeOB sequences

To determine the Gallionellaceae-related FeOB community composition associated with bulk water and sediments from mining-impacted and non-impacted streams, a total of 22 DGGE bands were excised from the gel, sequenced and analyzed phylogenetically. No chimeric sequence was detected.

**Figure 3:**
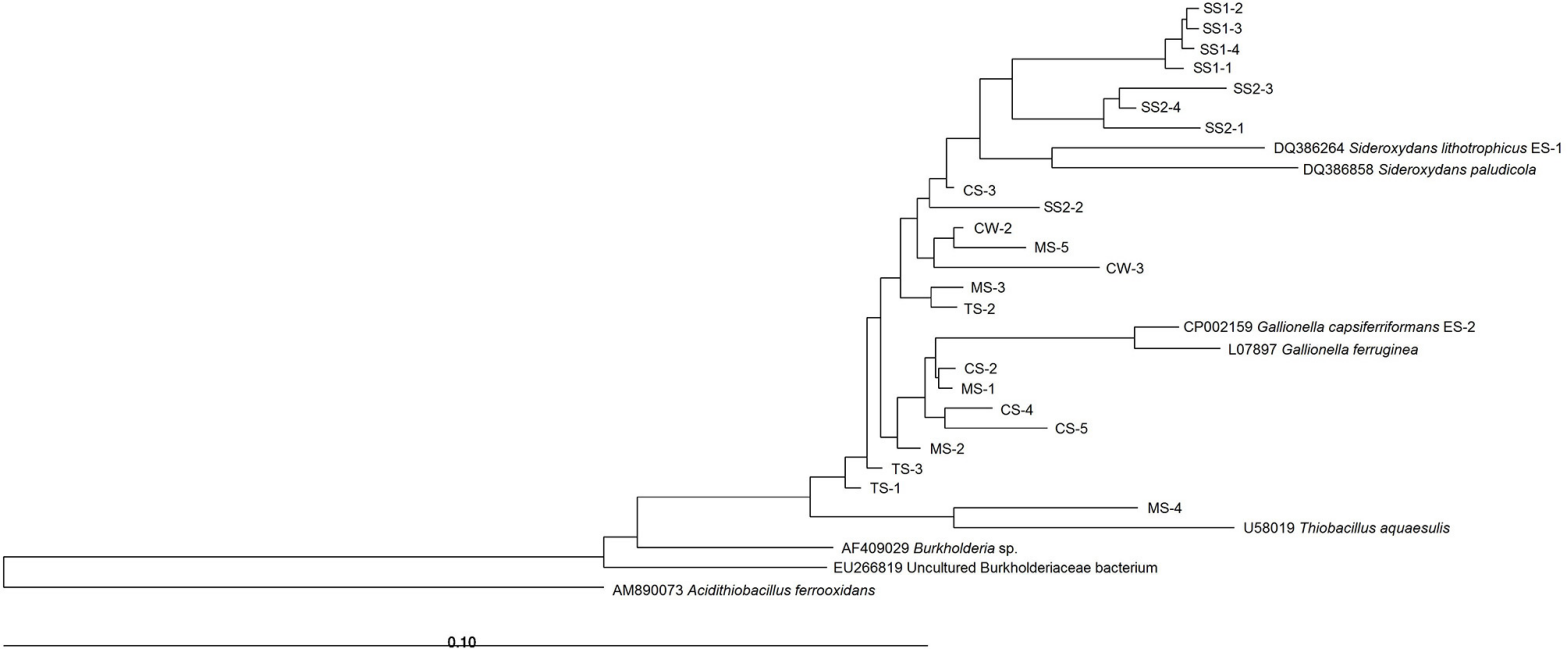
**Neighbor-joining tree of Gallionellaceae-related partial 16S rRNA gene sequences of DGGE bands**. CW, Carrapatos water; CS, Carrapatos sediment; MS, Mina sediment; SS1, Site 1 sediment; SS2, Site 2 sediment; TS, Tulipa sediment.

As expected, all sequences chosen for analysis were affiliated with the Betaproteobacteria class (Figure 3). The phylogenetic tree separated the samples in a way that was similar to the dendrogram analysis presented in Figure 2. Most sequences were affiliated with the neutrophilic FeOB genera *Gallionella* (7 bands) and *Sideroxydans* (14 bands). The *Sideroxydans*-related sequences were scattered throughout the samples, whereas *Gallionella*-related sequences were exclusive to mining-impacted sediments. Moreover, the MS-4 sequence was affiliated with the *Thiobacillus* genus (Figure 3), which contains also some iron-oxidizing species.

### Abundance of bacterial taxa related to gallionellaceae

**Figure 4:**
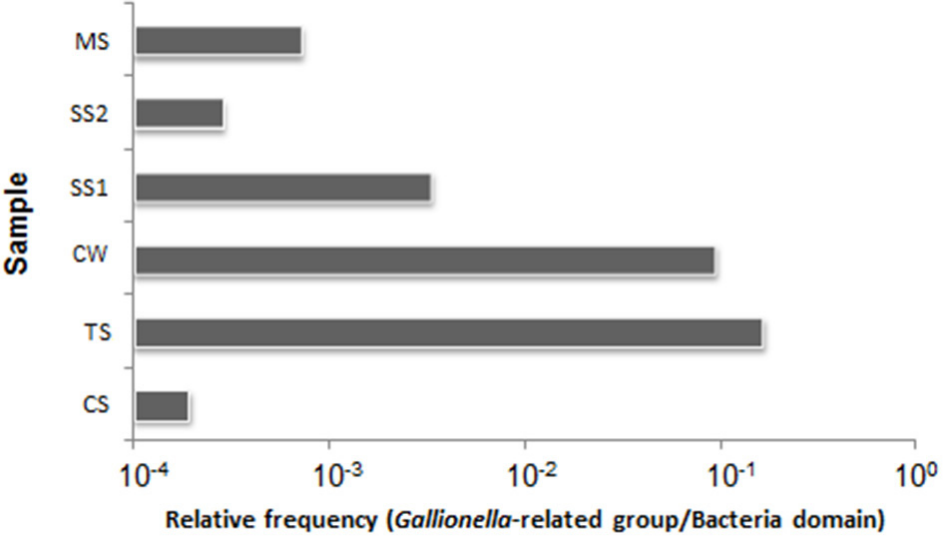
**Relative frequencies of the Gallionellaceae-related 16S rRNA gene copies in the total 16S rRNA genes of the Bacteria domain**. CW, Carrapatos water; CS, Carrapatos sediment; MS, Mina sediment; SS1, Site 1 sediment; SS2, Site 2 sediment; TS, Tulipa sediment.

To estimate the densities of Gallionellaceae-related taxa and of total Bacteria in each sample, 16S rRNA gene copy numbers were quantified by qPCR. The abundance of copies of 16S rRNA genes/g sediment or L of water, respectively, ranged from 1.16 × 10^4^ to 1.55 × 10^9^ for Bacteria, and 1.92 × 10^3^ to 4.42 × 10^5^ for Gallionellaceae-related taxa (Figure S3). The TS sample obtained from an environment with low concentrations of Fe, Mn, and Ni showed the highest relative abundance of Gallionellaceae-related 16S rRNA genes with a relative frequency of 1.65 × 10^−1^ (Figure 4). Oppositely, the CS sample obtained from the site with high concentrations of PO^3−^_4_-P, Mn, and Fe showed the lowest relative abundance of Gallionellaceae-related16S rRNA genes with a relative frequency of 1.92 × 10^−4^. The abundance of Bacteria in the CS sediment sample was three orders of magnitude higher than in the corresponding CW water sample, whereas the relative frequency of Gallionellaceae-related 16S rRNA genes was greater in the CW sample.

## Discussion

We explored the effect of anthropogenic activities on the phylogenetic diversity and the distribution of Gallionellaceae-related FeOB in streams located in the Iron Quadrangle, a region rich in ores such as iron and gold, which have been historically subjected to mining activities. The relationship between these neutrophilic bacteria with environmental physicochemical variables was also examined. Due to the region's natural metal richness, all the streams sampled showed a range of metals with concentrations depending directly on the proximity of each stream to the metal extraction ores. Our data demonstrated a ubiquitous distribution of betaproteobacterial taxa in all samples analyzed, independent of the metal concentration and trophic status of the streams.

The MS sample was closely correlated with a great number of variables, i.e., concentrations of inorganic nitrogen, As, Zn, and Cu. This result was congruent with the UPGMA cluster analysis of the Gallionellaceae-related FeOB band sequences, which showed the MS sample as the most dissimilar (Figures 1, 2). Thus, it appears that not only Fe, but also other variables such as inorganic nutrients, metals, and metalloids contribute to the diversity of Gallionellaceae-related FeOB. The DGGE fingerprinting patterns unveiled a distinct composition of Gallionellaceae-related FeOB in all sediment and water samples. The UPGMA cluster analysis of the DGGE bands revealed also a considerable dissimilarity among the samples, likely due to a large number of unique bands, indicating a diverse and specific community in each sample.

As shown in Figure 3, most sequences were affiliated with the *Gallionella* and *Sideroxydans* genera. Both genera are microaerophilic, lithotrophic Fe-oxidizers (Weiss et al., 2007). In our study, only mining-impacted sediments contained *Gallionella*-related sequences, suggesting a high degree of adaptation of *Gallionella* species to these environments impacted by anthropogenic activities. Samples from sediments of the non-impacted mining stream (SS1 and SS2) and from CW, whose metal and nutrient concentrations profile is close to those of the non-impacted sediments, showed only *Sideroxydans* sequences. As recently reported by Emerson et al. (2013), the *Gallionella* strain ES-2 genome harbors several resistance genes against different metals, such as As, Hg, Cd, Co, Ag, Zn, and Cu, differently from *Sideroxydans* strain ES-1. It seems that this characteristic can promote a competitive advantage for *Gallionella* in environments with high concentrations of potentially toxic metals and metalloids, which are typically found in mining-impacted sediments.

In the current study, the CW, SS1, and SS2 samples, which were only inhabited by the *Sideroxydans*-related sequences, were the least affected by the environmental variables analyzed, only displaying a slightly negative correlation with inorganic nitrogen. The absence of *Gallionella* in these samples points out the ability of *Sideroxydans* to occupy niches depleted in N, yielding a competitive advantage over taxa such as *Gallionella*, as suggested by Emerson et al. (2013). These authors revealed that *Sideroxydans* ES-1 has three clusters of *nif* genes, and is capable of growing in medium without a source of N, differently from *Gallionella ES-2*. Furthermore, Blöthe and Roden (2009) demonstrated that the majority of 16S rRNA gene sequences from a lithoautotrophic Fe(II)-oxidizing, nitrate-reducing enrichment culture were dominated by an autotrophic Fe(II) oxidizer related to *Sideroxydans lithotrophicus* (95% identical). Interestingly, a recent study on the succession among neutrophilic FeOB in streams also found species-specific ecological niches (Fleming et al., 2014). According to this work, members of the Gallionellales order dominated during the spring and were positively correlated with low organic carbon and steep redoxclines. In contrast, *Leptothrix ochracea* preferred the summer and high concentrations of complex organic carbon, high Fe and Mn contents and gentle redoxclines. We aimed at detecting Gallionellaceae-related FeOB, and the primers used were rather specific as only one out of 22 sequences obtained from the DGGE bands appeared to belong to another family (i.e., the Hydrogenophilaceae with the genus *Thiobacillus*).

The PCA analysis revealed that the concentrations of Fe, DO, and Mn affected the position of the CS sample in the biplot positively, but the position of the TS sample negatively. Interestingly, the highest and lowest relative frequencies of Gallionellaceae-related FeOB, observed in TS and CS samples, respectively, were associated with the lowest and highest concentrations of Fe. This inverse correlation may suggest that high abundance of Gallionellaceae-related FeOB is responsible for low Fe concentrations.

In conclusion, our data provide insight into the response of Gallionellaceae-related FeOB communities from tropical streams to anthropogenic activities. By itself, Fe does not appear to play a unique key role in shaping the diversity of neutrophilic FeOB communities in the investigated streams. Moreover, the data presented herein demonstrated that these communities form part of geosymbiotic systems, which likely appear to be significant not only for the biogeochemical cycling of Fe but also for the conversion of other metals, metalloids, and inorganic nutrients. Our data also reveal a different response among the Gallionellaceae, with *Gallionella* taking the advantage in environments impacted by anthropogenic activities over *Sideroxydans*. Hence, the absence of *Gallionella* sequences in environments remediated after mining activities might be used as an indicator of reaching the end point of remediation processes.

### Conflict of interest statement

The authors declare that the research was conducted in the absence of any commercial or financial relationships that could be construed as a potential conflict of interest.

## Supplementary material

Figure S1 **Sampled streams: (A) Carrapatos stream, (B) Site 1, (C) Site 2, (D) Tulipa stream, (E) Mina stream**.

Figure S2 **The Bacteria domain standard curve (A,C); the Gallionellaceae-related FeOB standard curve (B,D)**.

Figure S3 **The *Ct*-values of the Bacteria domain (A,C,E,G,I,K) and the Gallionellaceae-related (B,D,F,H,J,L) 16S rRNA gene amplifications from Carrapatos sediment (CS), Tulipa sediment (TS), Carrapatos water (CW), Site 1 sediment (SS1), Site 2 sediment (SS2), and Mina sediment (MS), respectively**.

Figure S4 **Denaturing gradient gel electrophoresis (DGGE) patterns showing Gallionellaceae-related FeOB sequences from the samples**. The bands analyzed by the BioNumerics software package are in black. The sequenced bands are highlighted with a scissors. CW, Carrapatos water; CS, Carrapatos sediment; MS, Mina sediment; SS1, Site 1 sediment; SS2, Site 2 sediment; TS, Tulipa sediment.

## References

[B1] Andrews S. C. Robinson A. K. Rodriguez-Quinones F. (2003). Bacterial iron homeostasis. FEMS Microbiol. Rev. 27, 215–237. 10.1016/S0168-6445(03)00055-X 12829269

[B2] Auernik K. S. Maezato Y. Blum P. H. Kelly R. M. (2008). The genome sequence of the metal-mobilizing, extremely thermoacidophilic archaeon *Metallosphaera sedula* provides insights into bioleaching-associated metabolism. Appl. Environ. Microbiol. 74, 682–692. 10.1128/AEM.02019-07 18083856 PMC2227735

[B3] Blöthe M. Roden E. E. (2009). Composition and activity of an autotrophic Fe(II)-oxidizing, nitrate-reducing enrichment culture. Appl. Environ. Microbiol. 75, 6937–6940. 10.1128/AEM.01742-09 19749073 PMC2772415

[B4] Cidu R. Biddaw R. Fanfani L. (2009). Impact of past mining activity on the quality of groundwater in SW Sardinia (Italy). J. Geochem. Explor. 100, 125–132 10.1016/j.gexplo.2008.02.003

[B5] Druschel G. K. Emerson D. Sutka R. Suchecki P. Luther G. W. III. (2008). Low-oxygen and chemical kinetic constraints on the geochemical niche of neutrophilic iron(II) oxidizing microorganisms. Geochim. Cosmochim. Acta 72, 3358–3370 10.1016/j.gca.2008.04.035

[B6] Emerson D. Field E. K. Chertkov O. Davenport K. W. Goodwin L. Munk C.. (2013). Comparative genomics of freshwater Fe-oxidizing bacteria: implications for physiology, ecology, and systematics. Front. Microbiol. 4:254. 10.3389/fmicb.2013.00254 24062729 PMC3770913

[B7] Emerson D. Fleming E. J. McBeth J. M. (2010). Iron-oxidizing bacteria: an environmental and genomic perspective. Annu. Rev. Microbiol. 64, 561–583. 10.1146/annurev.micro.112408.134208 20565252

[B8] Emerson D. Floyd M. M. (2005). Enrichment and isolation of iron-oxidizing bacteria at neutral pH. Methods Enzymol. 397, 112–123. 10.1016/S0076-6879(05)97006-7 16260287

[B9] Emerson D. Moyer C. (1997). Isolation and characterization of novel iron-oxidizing bacteria that grow at circumneutral pH. Appl. Environ. Microbiol. 63, 4784–4792. 9406396 10.1128/aem.63.12.4784-4792.1997 PMC168801

[B10] Emerson D. Moyer C. L. (2002). Neutrophilic Fe-oxidizing bacteria are abundant at the Loihi seamount hydrothermal vents and play a major role in Fe oxide deposition. Appl. Environ. Microbiol. 68, 3085–3093. 10.1128/AEM.68.6.3085-3093.2002 12039770 PMC123976

[B11] Emerson D. Weiss J. V. (2004). Bacterial iron oxidation in circumneutral freshwater habitats: findings from the field and the laboratory. Geomicrobiol. J. 21, 405–414 10.1080/01490450490485881

[B12] Felsenstein J. (1985). Confidence limits on phylogenies: an approach using the bootstrap. Evolution 39, 783–791 10.2307/2408678 28561359

[B13] Fleming E. J. Cetinic I. Chan C. S. Whitney King D. Emerson D. (2014). Ecological succession among iron-oxidizing bacteria. ISME J. 8, 804–815. 10.1038/ismej.2013.197 24225888 PMC3960539

[B15] Ghiorse W. C. (1984). Biology of iron-and manganese-depositing bacteria. Annu. Rev. Microbiol. 38, 515–550. 10.1146/annurev.mi.38.100184.002503 6388499

[B16] Hallbeck L. Pedersen K. (1995). Benefits associated with the stalk of *Gallionella ferruginea*, evaluated by comparison of stalk-forming and a non-stalk forming strain and biofilm studies *in-situ*. Microbial. Ecol. 30, 257–268. 10.1007/BF00171933 24185563

[B17] Hallbeck L. Stahl F. Pedersen K. (1993). Phylogeny and phenotypic characterization of the stalk-forming and iron-oxidizing bacterium *Gallionella ferruginea*. J. Gen. Microbiol. 139, 1531–1535. 10.1099/00221287-139-7-1531 8371116

[B18] Hammer Ø. Harper D. A. T. Ryan P. D. (2001). PAST: paleontological statistics software package for education and data analysis. Palaeontol. Electronic. 4, 1–9 Available online at: http://palaeo-electronica.org/2001_1/past/issue1_01.htm

[B19] Harrison A. P. (1984). The acidophilic thiobacilli and other acidophilic bacteria that share their habitat. Annu. Rev. Microbiol. 38, 265–292. 10.1146/annurev.mi.38.100184.001405 6388492

[B20] Hedrich S. Schlömann M. Barrie Johnson D. (2011). The iron-oxidizing proteobacteria. Microbiology 157, 1551–1564. 10.1099/mic.0.045344-0 21511765

[B21] Huber T. Faulkner G. Hugenholtz P. (2004). Bellerophon: a program to detect chimeric sequences in multiple sequence alignments. Bioinformatics 20, 2317–2319. 10.1093/bioinformatics/bth226 15073015

[B22] Johnson D. B. Hallberg K. B. (2003). The microbiology of acidic mine waters. Res. Microbiol. 154, 466–473. 10.1016/S0923-2508(03)00114-1 14499932

[B23] Jolliffe I. T. (2002). Principal Component Analysis. New York, NY: Springer.

[B24] Koroleff F. (1976). Determination of nutrients, in Methods of Seawater Analysis, ed Grasshoff K. (New York, NY: VerlChemieWeinhein), 117–181.

[B25] Leduc L. G. Ferroni G. D. (1994). The chemolithotrophic bacterium *Thiobacillus ferrooxidans*. FEMS Microbiol. Rev. 14, 103–119 10.1111/j.1574-6976.1994.tb00082.x

[B26] Lin C. Larsen E. I. Nothdurft L. D. Smith J. J. (2012). Neutrophilic, microaerophilic Fe(II)-oxidizing bacteria are ubiquitous in aquatic habitats of a subtropical australian coastal catchment (ubiquitousFeOBin catchment aquatic habitats). Geomicrobiol. J. 29, 76–87 10.1080/01490451.2010.523446

[B27] Lovley D. R. (2000). Fe(III) and Mn(IV) reduction, in Environmental Microbe–Metal Interactions, ed Lovley D. R. (Washington, DC: ASM Press), 3–30 10.1128/9781555818098.ch1

[B28] Ludwig W. Strunk O. Westram R. Richter L. Meier H. Yadhukumar. (2004). ARB: a software environment for sequence data. Nucleic Acids Res. 32, 1363–1371. 10.1093/nar/gkh293 14985472 PMC390282

[B29] Lutgens F. K. Tarbuck E. J. (2000). Essentials of Geology. New York, NY: Prentice Hall.

[B30] Mackereth J. F. H. Heron J. Talling J. F. (1978). Water analysis: some revised methods for limnologists. Freshw. Biol. Assoc. 36, 117–121.

[B31] McAllister M. L. Milioli G. (2000). Mining sustainably: opportunities for Canada and Brazil. Miner. Energy 15, 3–14 10.1080/14041040009362553

[B32] McBeth J. M. Little B. J. Ray R. I. Farra K. M. Emerson D. (2011). Neutrophilic iron-oxidizing “Zetaproteobacteria” and mild steel corrosion in nearshore marine environments. Appl. Environ. Microbiol. 77, 1405–1412. 10.1128/AEM.02095-10 21131509 PMC3067224

[B33] McCune B. Grace J. B. (2002). Analysis of Ecological Communities. Gleneden Beach: MjM.

[B34] Muyzer G. De Waal E. C. Uitterlinden A. G. (1993). Profiling of complex microbial populations by denaturing gradient gel electrophoresis analysis of polymerase chain reaction-amplified genes coding for 16S rRNA. Appl. Environ. Microbiol. 59, 695–700. 7683183 10.1128/aem.59.3.695-700.1993 PMC202176

[B35] Pronk J. T. Johnson D. B. (2002). Oxidation and reduction of iron by acidophilic bacteria. Geomicrobiol. J. 10, 153–171 10.1080/01490459209377918

[B36] Pruesse E. Quast C. Knittel K. Fuchs B. M. Ludwig W. Peplies J.. (2007). SILVA: a comprehensive online resource for quality checked and aligned ribosomal RNA sequence data compatible with ARB. Nucleic Acids Res. 35, 7188–7196. 10.1093/nar/gkm864 17947321 PMC2175337

[B37] Raskin L. Stromley J. M. Rittmmann B. E. Stahl D. A. (1994). Group-specific 16S rRNA hybridization probes to describe natural communities of methanogens. Appl. Environ. Microbiol. 60, 1232–1240. 7517128 10.1128/aem.60.4.1232-1240.1994 PMC201464

[B38] Reis M. P. Barbosa F. A. R. Chartone-Souza E. Nascimento A. M. A. (2013). The prokaryotic community of a historically mining-impacted tropical stream sediment is as diverse as that from a pristine stream sediment. Extremophiles 17, 301–309. 10.1007/s00792-013-0517-9 23389654

[B39] Smedley P. L. Kinniburgh D. G. (2002). A review of the source, behavior and distribution of arsenic in natural waters. Appl. Geochem. 17, 517–568 10.1016/S0883-2927(02)00018-5

[B40] Sogaard E. G. Aruna R. Abraham-Peskir J. Bender Koch C. (2001). Conditions for biological precipitation of iron by *Gallionella ferruginea* in a slightly polluted ground water. Appl. Geochem. 16, 1129–1137 10.1016/S0883-2927(01)00014-2

[B41] Thomas J. E. Smart R. S. C. Skinner W. M. (2000). Kinetic factors for oxidative and non-oxidative dissolution of iron sulfides. Min. Eng. 13, 1149–1159 10.1016/S0892-6875(00)00098-4

[B42] Varejão E. V. V. Bellato C. R. Fontes M. P. F. Mello J. W. V. (2011). Arsenic and trace metals in river water and sediments from the southeast portion of the Iron Quadrangle, Brazil. Environ. Monit. Assess. 172, 631–642. 10.1007/s10661-010-1361-3 20238242

[B43] Wang J. Muyzer G. Bodelier P. Laanbroek H. J. (2009). Diversity of iron oxidizer in wetland soils revealed by novel 16S rRNA primers targeting *Gallionella*-related bacteria. ISME J. 3, 715–725. 10.1038/ismej.2009.7 19225553

[B44] Wang J. Vollrath S. Behrends T. Bodelier P. L. E. Muyzer G. Meima-Franke M.. (2011). Distribution and diversity of *Gallionella*-like neutrophilic iron oxidizers in a tidal freshwater marsh. Appl. Environ. Microbiol. 77, 2337–2344. 10.1128/AEM.02448-10 21317256 PMC3067411

[B45] Weiss J. V. Rentz J. A. Plaia T. Neubauer S. C. Merrill-Floyd M. Lilburn T. (2007). Characterization of neutrophilicFe(II)-oxidizing bacteria isolated from the rhizosphere of wetland plants and description of *Ferritrophicum radicicola* gen. nov. sp. nov., and *Sideroxydans paludicola* sp. nov. Geomicrobiol. J. 24, 559–570 10.1080/01490450701670152

