# Supplementary figures and images for: The influence of human settlement on the distribution and diversity of iron-oxidizing bacteria belonging to the Gallionellaceae in tropical streams
Source: Front Microbiol. 2014 Nov 24;5:630. doi: 10.3389/fmicb.2014.00630 (PMC4241827; doi:10.3389/fmicb.2014.00630)

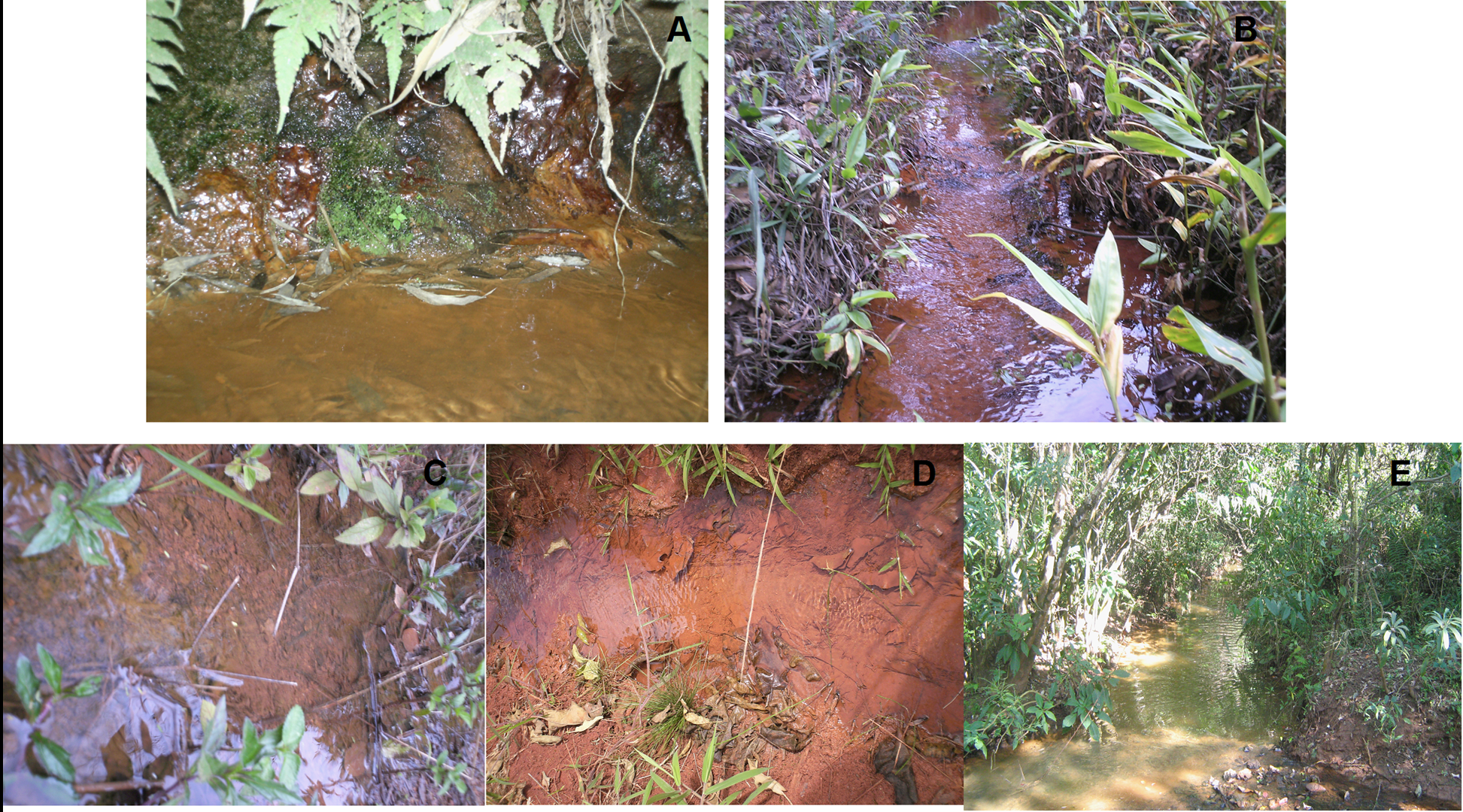

Supplement: Figure S1 — Sampled streams: (A) Carrapatos stream, (B) Site 1, (C) Site 2, (D) Tulipa stream, (E) Mina stream. [file Image1.TIF]

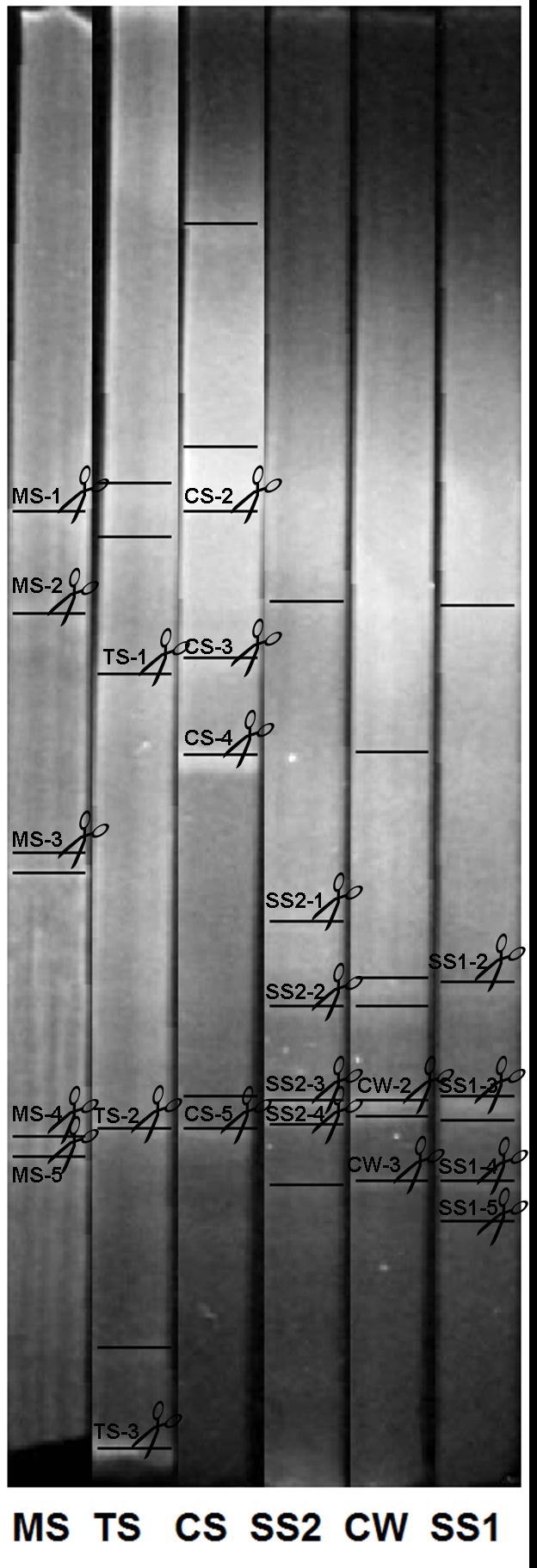

Supplement: Figure S4 — Denaturing gradient gel electrophoresis (DGGE) patterns showing Gallionellaceae-related FeOB sequences from the samples. The bands analyzed by the BioNumerics software package are in black. The sequenced bands are highlighted with a scissors. CW, Carrapatos water; CS, Carrapatos sediment; MS, Mina sediment; SS1, Site 1 sediment; SS2, Site 2 sediment; TS, Tulipa sediment. [file Image4.TIF]
